# Biopolymers Take Center Stage in Wound Healing Advancements

**DOI:** 10.3390/pharmaceutics16060755

**Published:** 2024-06-03

**Authors:** Gorka Orive, Martín Federico Desimone

**Affiliations:** 1 NanoBioCel Research Group, School of Pharmacy, University of the Basque Country (UPV/EHU), 01006 Vitoria-Gasteiz, Spain; 2 NanoBioCel Research Group, Bioaraba, 01009 Vitoria-Gasteiz, Spain; 3 Biomedical Research Networking Centre in Bioengineering, Biomaterials and Nanomedicine (CIBER-BBN), 01006 Vitoria-Gasteiz, Spain; 4 University Institute for Regenerative Medicine and Oral Implantology—UIRMI (UPV/EHU-Fundación Eduardo Anitua), 01007 Vitoria-Gasteiz, Spain; 5 Universidad de Buenos Aires, Facultad de Farmacia y Bioquímica, Departamento de Ciencias Químicas, Cátedra de Química Analítica Instrumental, Buenos Aires 1113, Argentina; 6 Consejo Nacional de Investigaciones Científicas y Técnicas, Instituto de Química y Metabolismo del Fármaco (IQUIMEFA), Universidad de Buenos Aires, Buenos Aires 1113, Argentina

The human body possesses a remarkable ability to heal itself from injuries. However, chronic wounds may disrupt this natural process, causing significant health problems and impacting millions of people globally. Effective treatments for chronic wounds are a pressing medical need, and biopolymers have emerged as a promising class of materials with the potential to revolutionize wound healing strategies.

Biopolymers, as naturally occurring polymers derived from biological sources, offer unique advantages for wound healing applications. Their biocompatibility, biodegradability, and structural versatility make them ideal candidates for developing novel wound dressings, drug delivery systems, and tissue engineering scaffolds. Scientific research underscores the exciting possibilities of natural polymers in the field of biomedicine. Further exploration into the composition and methods for preparing these biopolymer-based materials reveals their potential for application throughout every critical stage of wound healing [1].

The limitations of traditional wound management techniques, such as skin grafting, have driven the development of innovative biocompatible polymeric materials. In this sense, natural and synthetic biopolymers offer significant advantages compared to conventional methods. These biopolymers address challenges associated with donor site availability, immune rejection, and immunological responses, making them promising candidates for dermal regeneration [2].

Current wound healing strategies have well-established limitations. The focus needs to shift beyond simply achieving macroscopic closure of the wound. The importance of addressing the microscopic abnormalities that contribute to chronic wounds is becoming increasingly evident. This shift necessitates the development of new, integrated therapies that combine drugs, biomolecules, and biomaterials to achieve faster and more effective wound healing [3].

Chronic wounds not only pose a significant health risk but also severely impact patients’ quality of life. Pain, discomfort, and delayed healing can have a profound psychological toll. Selecting the appropriate wound dressing is crucial for supporting and accelerating the healing process [4]. Hydrogels, with their high water content, moist environment, biocompatibility, and biodegradability, have emerged as a leading material for wound dressings. Their resemblance to dermal tissues and ability to deliver therapeutic agents directly to the wound site make them particularly promising.

The ideal wound dressing should not only create a suitable environment for healing but also adapt to the specific needs of the wound throughout the healing process. Various natural polymers, such as chitosan, collagen, and alginate, are being used to develop advanced wound dressings with properties like biocompatibility, biodegradability, and similarity to the human extracellular matrix (ECM). These dressings can take various forms, including hydrogels, nanofibers, sponges, and films, each offering unique advantages for different wound types [5].

However, some challenges remain, such as limitations in adherence, opacity, and controlled drug delivery. The development of “smart” wound dressings that can monitor wound healing progress and release therapeutic agents accordingly is a promising area for future research [6]. One promising avenue for addressing these challenges lies in the development of “smart” wound dressings [7]. These advanced materials hold the potential to revolutionize wound care by not only creating an optimal environment for healing but also actively monitoring progress and delivering therapeutic agents as needed. Ideally, wound dressings should adapt to the changing wound microenvironment, providing the specific support needed at each phase of healing. 

Hydrogel-based dressings have emerged as powerful tools, offering favorable biological properties and the potential for active intervention during the healing process. Recent research focuses on developing “smart” hydrogels and 3D-printed scaffolds that can regulate the release of therapeutic agents [8]. These materials respond to both external stimuli, such as light, and internal changes within the wound itself, like temperature, pH, and the presence of specific molecules. This allows for targeted and controlled drug delivery, optimizing treatment based on the real-time needs of the wound [9]. 

The future of wound care is even more exciting with the exploration of “sense-and-treat” dressings. These intelligent materials integrate built-in sensors or sensing molecules with responsive hydrogels. This enables real-time monitoring of wound conditions, allowing for a tailored and responsive therapeutic approach. By combining active treatment with continuous monitoring, “smart” wound dressings have the potential to significantly improve healing outcomes and reduce the burden on healthcare systems [10].

Biopolymers are not only valuable for developing wound dressings but also hold immense potential for tissue engineering applications. Their biocompatibility and biodegradability make them ideal building blocks for scaffolds that can support tissue regeneration. These biopolymers can be derived from various natural sources and engineered to mimic the properties of specific tissues, such as skin, bone, or nerves. Biopolymer-based composites offer even greater potential, combining the properties of different materials to create scaffolds with tailored functionalities [11]. However, further research is needed to overcome challenges associated with composite design and functionality and fully realize the potential of biopolymers in tissue engineering.

This special edition of *Pharmaceutics*, titled “Biopolymer Materials for Wound Healing, Second Edition,” builds upon the foundation laid in the first edition by showcasing the latest advancements in this rapidly evolving field. Biopolymers, naturally occurring polymers derived from biological sources, offer unique advantages for wound healing applications. Their biocompatibility, biodegradability, and structural versatility make them ideal candidates for developing novel wound dressings, drug delivery systems, and tissue engineering scaffolds. This collection of articles highlights different aspects of biopolymer-based wound healing technologies. It explores how researchers are harnessing the properties of biopolymers to create innovative solutions for different wound healing challenges. From facilitating controlled drug delivery to modulating the cellular environment and mitigating oxidative stress, biopolymers are shaping the future of wound care.

The first article investigates a low-molecular-weight hydrogel (LMWG) for topical delivery of copper salts in wound healing. The authors demonstrate that the hydrogel transforms into a self-assembled fibrillar network (SAFiN) upon drug loading, enhancing its mechanical strength. In vivo studies showed faster wound closure compared to controls, suggesting promise for this approach.

The second article explores the use of freeze-drying to preserve bFGF growth factor for chronic wound treatment. The authors identify trehalose as an effective cryoprotectant, stabilizing the coacervate particles and preserving bFGF activity. This finding paves the way for a more robust topical delivery system for growth factors.

The third article reviews polymeric films for wound dressings, fabricated using the solvent casting technique. This cost-effective method allows for the creation of biocompatible and easily applicable wound dressings with tailored properties. The article explores critical factors influencing the performance of these dressings.

Bacterial cellulose (BC) is a versatile biomaterial with potential applications in wound healing. The fourth article reviews the use of BC in various formats, including membranes, films, and nanofibers. The authors also discuss the development of novel antimicrobial BCs and drug delivery systems based on BC.

Curcumin, a natural compound derived from turmeric, possesses excellent wound healing properties. However, its clinical use is limited by its poor water solubility and rapid metabolism. The fifth article reviews recent advances in curcumin delivery systems for wound healing, including hydrogels, films, and nanoformulations. These systems enhance curcumin’s therapeutic efficacy by improving its solubility and bioavailability.

Hydrogels offer a promising platform for controlled delivery of therapeutic agents in wound healing. The sixth article explores the potential of hydrogels for in situ delivery of antioxidants, growth factors, and antibiotics to promote wound healing. The article also discusses the role of hydrogels in modulating macrophage polarization, a critical factor in chronic wound repair.

Chronic wounds are often associated with oxidative stress. The seventh article reviews the use of natural polymers with antioxidant properties to create tissue regeneration microenvironments for wound healing. This approach offers a promising strategy to combat oxidative stress and promote healing in chronic wounds.

In conclusion, this Special Issue presents a comprehensive overview of recent advancements in wound healing technologies. The articles highlight the potential of novel drug delivery systems, biocompatible materials, and antioxidant therapies to improve wound healing outcomes. These innovative approaches offer promising avenues for the development of more effective wound care treatments.
